# A Comparison Study on the Arsenate Adsorption Behavior of Calcium-Bearing Materials

**DOI:** 10.3390/ma12121936

**Published:** 2019-06-16

**Authors:** Han Wang, Hong Zhu

**Affiliations:** College of Materials Science and Engineering, Nanjing Tech University, Nanjing 210009, China; hwang1227@163.com

**Keywords:** calcium-bearing materials, As(V), adsorption properties, adsorption mechanism

## Abstract

The calcium-bearing adsorbents are widely used in the treatment of arsenic-containing wastewater due to their excellent treatment effect and economy. In order to obtain high-efficient adsorbents for arsenate (As(V)) removal, the adsorption behavior of calcium oxide (CaO), calcium fluoride (CaF_2_) and calcium carbonate (CaCO_3_) on As(V) in aqueous solution at different concentrations were explored. The adsorption mechanism was also explored based on surface characteristics: morphology, specific surface area, as well as their effective calcium content. Not only that, the chemical stability of these materials was further studied. Results exhibited that the As(V) removal capability of these materials is in the following order, CaO > CaF_2_ > CaCO_3_. When CaO served as an absorbent, As(V) with initial concentration of 0.2 mg/L can be reduced to 0.383 × 10^−3^ mg/L in 10 min. Moreover, the capabilities of CaO, CaF_2_ and CaCO_3_ for removing As(V) are positively correlated with their effective calcium content in aqueous solution, which provide the basis for selecting calcium-bearing materials with excellently comprehensive properties for the field of As(V) removal in aqueous solution. What’s more, all three materials exhibit great chemical stability after adsorption of As(V).

## 1. Introduction

With the rapid development of industry and agriculture, the problem of water pollution caused by arsenic is becoming increasingly serious [1,2,3]. Arsenic is a carcinogen with a wide range of biological effects [4,5]. Long-term drinking of arsenic-containing water will lead to a series of health problems such as melanosis, keratosis and cancer, etc. [6,7,8]. The hygienic standards of highest concentration of arsenic in drinking water are strictly defined by the World Health Organization (WHO), Japan, America and China (less than 0.01 mg/L) [9]. Not only that, the highest concentration of arsenic in effluent industrial wastewater in China is also strictly defined (less than 0.5 mg/L). There have been many reports about arsenic contamination in water bodies over the years [10,11]. Arsenic pollution in water is widespread in more than 70 countries, especially in Bangladesh, India and China [12,13]. Therefore, limiting the concentration of arsenic in water is extremely urgent and significant to reduce the harm of arsenic. Generally speaking, the treatment methods for arsenic pollution in water can be divided into the chemical precipitation method, ion exchange method, membrane separation method, electrochemical method, and adsorption method [14,15,16]. Compared with the other methods, the adsorption method is widely used because of the advantages of a wide source of raw materials, easy operation and high efficiency, etc.

Among many adsorbents, calcium-bearing adsorbents are widely used in the treatment of arsenic-containing wastewater due to their excellent treatment effect and economy, e.g., by calcite and lime [17,18]. The adsorption mechanism of arsenic by calcium-containing adsorbents is mainly the combination of calcium arsenate between arsenate anions and calcium ions (Ca^2+^). As Bothe et al. [19] reported, the main reaction product was calcium arsenate using lime as an adsorbent to remove arsenic. Although there have been many studies on the treatment of arsenic-containing wastewater by calcium-bearing materials, the arsenic removal efficiency of calcium-bearing materials with different types and particle sizes is significantly different. Furthermore, there also a lack of systematical comparison reports on their arsenic-removal performance. More than that, the kinetic process and mechanism of arsenic removal on different calcium-bearing materials are rarely discussed. 

In order to obtain high-efficient calcium-bearing adsorbents for arsenate (As(V)) removal and compare their As(V) removal mechanisms, the As(V) adsorption behaviors in water solution of CaO, CaF_2_ and CaCO_3_ were studied. Not only were the capabilities of removing As(V) for these materials compared according to removal rates and adsorption rates, but also As(V) removal mechanisms were analyzed based on calculation of the material’s crystal structures and specific surface areas. The study will provide the basis for selecting calcium-bearing materials with excellently comprehensive properties for the field of arsenic removal in aqueous solution.

## 2. Materials and Methods

### 2.1. Materials

Calcium fluoride (CaF_2_) and calcium carbonate (CaCO_3_) were purchased from Sinopharm Chemical Reagent Co., Ltd. and Beijing Huaweiruike Chemical Co., Ltd., respectively.

Calcium oxide (CaO, with a certain thickness on the surface of CaCO_3_) was obtained by calcining and decomposing calcium carbonate (CaCO_3_), and CaCO_3_ was used to prepare CaO samples is the same substance as CaCO_3_ used in this study.

### 2.2. Experimental Methods

#### 2.2.1. Preparation of Arsenic-Containing Aqueous Solution

Na_3_AsO_4_·12H_2_O (analytical grade, 5.6576 g) was dissolved into deionized water using a volumetric flask of 1000 mL. A simulated stock solution of 1000 mg/L As(V) was prepared. The as-prepared arsenic stock solution was diluted to the required concentrations (0.2 and 30 mg/L) with deionized water, and the pH value was adjusted to 7.0 ± 0.1 by hydrochloric acid (HCl, analytical grade) and Sodium hydroxide (NaOH, analytical grade) using pH meter (PHS-25, Shanghai Yidian Scientific Instrument co., Ltd., Shanghai, China).

#### 2.2.2. Adsorption Experiment

Each adsorbent (0.2 g) was separately added to glass containers prefilled with 20 mL arsenic-containing aqueous solution. The obtained suspensions were mixed in a water bath thermostatic oscillator for a certain period of time at a pH of 7 and temperature of 25 °C. Then, supernatants were filtered through a membrane of 0.22 µm, and As(V) concentrations were detected by AFS-230E double-channel hydride generation atomic fluorescence photometer.

The adsorption quantity of the adsorbents was calculated according to the Equation (1):

(1) $$q_{e}=\left(C_{0}-C_{e}\right)V/m$$

where $q_{e}$ is the adsorption quantity at adsorption equilibrium, mg/g; V is the volume of arsenic-containing solution, L; m is the adsorbent dosage, g; $C_{0}$ and $C_{e}$ are the initial and equilibrium concentrations of arsenic in the solution, respectively, mg/L.

### 2.3. Characterizations of Calcium-Bearing Materials

The decomposition temperature of CaCO_3_ was analyzed by thermogravimetric analysis (TGA, METTLERTGA2, Mettler Toledo, Zurich, Switzerland). The morphologies and particle sizes of the adsorbents were observed by scanning electronic microscope (SEM, JSM-6510, Tokyo, Japan). The specific surface areas of the adsorbents were detected by Brunauer–Emmett–Teller (BET, ASAP2460, Micromeritics, Atlanta, Georgia, America). The crystal structures of materials were identified by X-ray diffraction (XRD, Smartlab (3 kw), Rigaku Corporation, Tokyo, Japan), testing conditions: room temperature, voltage of 40 kV and current of 30 mA, Cu K alpha, two-theta testing angle range: 5–80°, testing speed: 5 °/min.

## 3. Results and Discussion

### 3.1. Characteristics of CaO

**Figure 1:**
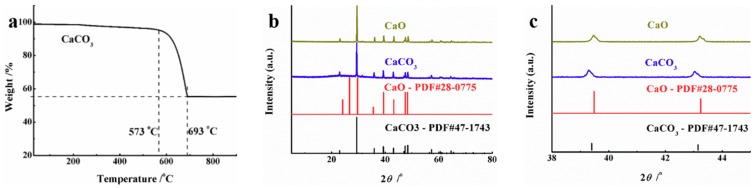
(**a**) Thermogravimetric analysis (TGA) curve of CaCO_3_, (**b**) and (**c**) X-ray diffraction (XRD) patterns of CaO and CaCO_3_ at different two-theta testing angle ranges of 5–80° and 38–45°.

The CaO used in this study was obtained by decomposition of CaCO_3_ at high temperature. Therefore, the initial decomposition temperature of CaCO_3_ need to be explored, and the TGA of CaCO_3_ was carried out under air atmosphere with a heating rate of 5 °C/min. Figure 1a shows that the initial decomposition temperature of CaCO_3_ was about 573 °C, the weightlessness rate of CaCO_3_ was 13% at 633 °C, and completely weightless at 693 °C. In order to prepare CaO for the experiment, CaCO_3_ was put into a muffle furnace for burning at 633 °C, and the other burning conditions were the same as testing of TGA. In addition, CaO mass fraction was 15.9% in the obtained product. As shown in Figure 1b, the CaCO_3_ used in this study was a calcite crystal. The peaks of CaCO_3_ at 23.1°, 29.4°, 36.0° and 39.4° are corresponding to the crystal face of (1, 0, 2), (4 1 0), (0 1, 1) and (1, 1–3), respectively. Combining Figure 1b and c, the peaks at 39° and 43° of prepared CaO had a slight shift compared with pure CaCO_3_, which indicate that the samples cauterized at 633 °C had two phases of CaCO_3_ and CaO, thus proving that CaO was synthesized on the surface of CaCO_3_ with a certain thickness.

### 3.2. The As(V) Removal Capacities of Three Calcium-Bearing Materials at Different Initial Concentrations 

**Figure 2:**
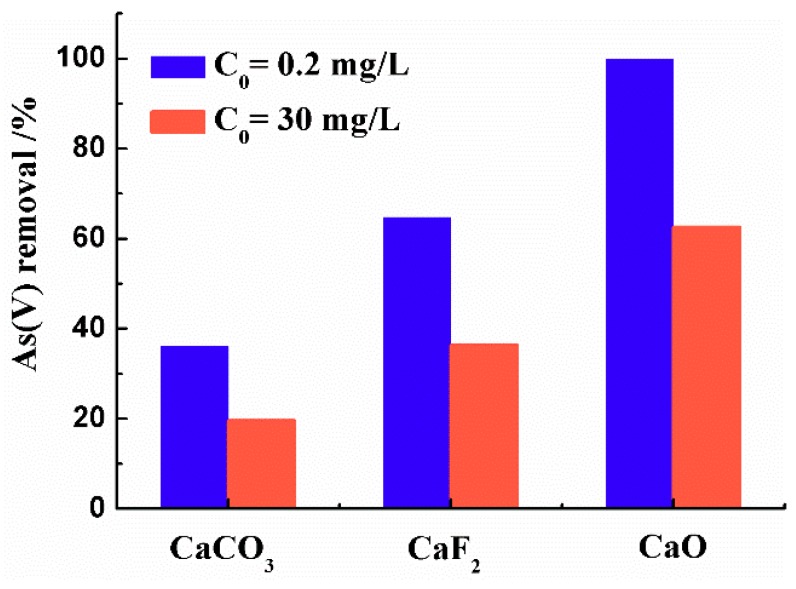
Arsenate (As(V)) adsorption capacities of three calcium-bearing materials at the As(V) initial concentration of 0.2 mg/L and 30 mg/L. (The dosage of calcium-containing materials was 10 g/L, the adsorption time was 24 h, the pH value of aqueous solution was 7, and the reaction temperature was 25 °C.)

The As(V) removal rates with different As(V) initial concentrations of three calcium-bearing materials after 24 h are shown in Figure 2. It can be found that there were significant differences in the As(V) removal capacities of the three calcium-bearing materials. Especially, CaO exhibited the strongest removal ability, followed by CaF_2_ and CaCO_3_. The removal rates of As(V) with initial concentration of 0.2 mg/L for CaO, CaF_2_ and CaCO_3_ were 99.9%, 68.0% and 36.1%, respectively. When the initial concentration of As(V) was increased to 30 mg/L, the removal rates of As(V) for the three calcium-bearing materials were all significantly reduced, which were 62.6%, 36.4% and 20.3%, respectively. Compared with initial concentration of 0.2 mg/L, the reduction rates of adsorption capacities for the CaO, CaF_2_ and CaCO_3_ were 37.3%, 46.5% and 43.8%, respectively, under initial concentration of 30 mg/L. Among them, CaO with the best adsorption performance decreased the least.

### 3.3. Effects of Adsorption Time on the As(V) Removal Capacities of Three Calcium-Bearing Materials

**Figure 3:**
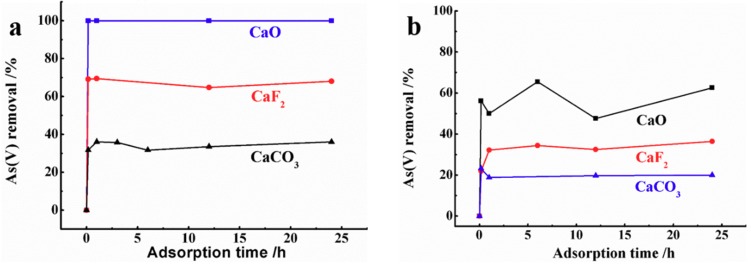
As(V) adsorption capacities of three calcium-bearing materials as function of adsorption time at (**a**) 0.2 mg/L and (**b**) 30 mg/L. (The dosage of calcium-containing materials was 10 g/L, the pH value of aqueous solution was 7, and the reaction temperature was 25 °C.)

The removal rates of As(V) with initial concentration of 0.2 mg/L for the three calcium-bearing materials at different adsorption times are shown in Figure 3a. It can be found that, CaO, CaF_2_, and CaCO_3_ can reach the adsorption equilibrium at 10 min, and the removal rates of As(V) were 99.8%, 69.1% and 31.8%, respectively.

The removal rates of As(V) with initial concentration of 30 mg/L for the three calcium-bearing materials at different adsorption times are shown in Figure 3b. By comparing the effects of adsorption time on the As(V) removal capacities at low (Figure 3a) and high (Figure 3b) initial concentrations, it can be concluded that increasing the initial concentration has a different influence on the time to reach the adsorption equilibrium for the three calcium-bearing materials. As for CaO, the time of reaching adsorption equilibrium has no significant change. As shown in Figure 3b, when CaO was adsorbed for 10 min, the removal rate of As(V) reached 56.2%. The free calcium content of CaO in aqueous solution is as high as 42.00 × 10^−2^ mmol, which mainly forms calcium arsenate precipitation to remove As(V). But the formed calcium arsenate precipitate is unstable [20], resulting in a wide fluctuation range of As(V) removal rate with the prolonging of adsorption time. The time for CaF_2_ to reach adsorption equilibrium was slightly extended. When the adsorption time increased from 10 min to 60 min, the removal rate of As(V) increased slowly from 21.9% to 32.2%, and the CaF_2_ reached adsorption equilibrium. The time for CaCO_3_ to reach adsorption equilibrium has no significant change. When CaCO_3_ was adsorbed for 10 min, the removal rate of As(V) reached 23.2%. 

In general, when the initial concentration of As(V) increased from 0.2 mg/L to 30 mg/L, both CaO and CaCO_3_ can reach adsorption equilibrium in 10 min, and the prolonged time has little effect on the removal rate of As(V). Compared with CaO and CaCO_3_, the time for CaF_2_ to reach adsorption equilibrium is slightly prolonged. What’s more, using CaO as an adsorbent to treat As(V) at the concentration of 0.2 mg/L in aqueous solution can meet the requirement of arsenic limit (0.01 mg/L) of the China sanitary standard for drinking water, while CaF_2_ used as an adsorbent to treat As(V) of 0.2 mg/L in aqueous solution can meet the requirement of arsenic limit (0.1 mg/L) of China water quality standard for farmland irrigation (category II).

### 3.4. Mechanism Analysis

The particle size, morphology and specific surface area are very important surface characteristics for adsorbents. Moreover, the main mechanism for the removal of As(V) by calcium-bearing materials is the formation of insoluble calcium arsenate salt generated by arsenate anions and calcium ions [21]. In order to demonstrate the mechanism of As(V) removal by calcium-bearing materials, the particle morphologies, specific surface areas and effective calcium content in aqueous solution of the three materials were compared. Especially, the effective calcium content of these materials is the sum of the surface calcium content and the free calcium content in the aqueous solution. 

#### 3.4.1. Analysis of Particle Morphologies and Specific Surface Areas

**Figure 4:**
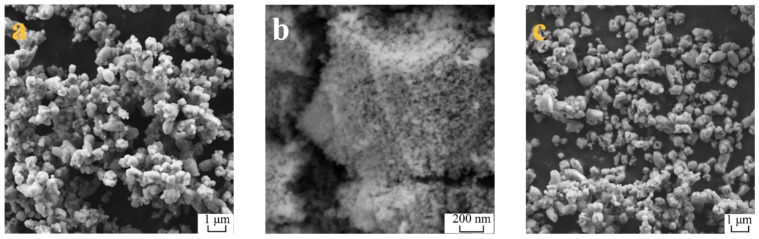
SEM images of (**a**) CaO, (**b**) CaF_2_ and (**c**) CaCO_3_.

SEM images of the three calcium-bearing materials before adsorption of As(V) are shown in Figure 4. Figure 4a and c exhibit CaCO_3_ and CaO, respectively. Both CaCO_3_ and CaO are spherical particles, and their particle sizes have no significatly change before and after burning, remaining within the range of 0.5~1 μm. Figure 4b shows that CaF_2_ has fine particle smaller than 50 nm.

**Figure 5:**
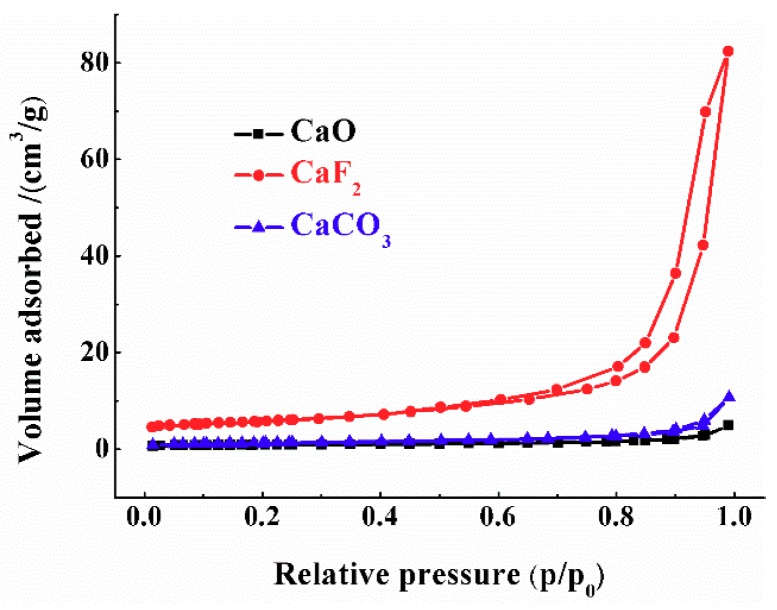
N_2_ adsorption and desorption isotherms of three calcium-bearing materials.

The nitrogen adsorption−desorption isotherms of the three calcium-containing materials are shown in Figure 5, from which we can get that the specific surface areas of these materials, is in the following order, CaF_2_ (21.05 m^2^/g) > CaCO_3_ (4.62 m^2^/g) > CaO (3.32 m^2^/g). 

As(V) adsorption capacity of the three calcium-bearing materials at the initial concentration of 30 mg/L is in the following order, CaO (2.00 mg/g) > CaF_2_ (1.10 mg/g) > CaCO_3_ (0.63 mg/g). Taking both morphological observation and specific surface areas into consideration, CaF_2_ with smallest particle size and the largest specific surface area did not show the best adsorption capacity on As(V); The specific surface areas of CaO and CaCO_3_ were similar, but the adsorption capacity of As (V) was significantly different. Therefore, we can draw a preliminary conclusion that the morphology and specific surface area were not the significant determinants for the As(V) removal abilities of the three calcium-bearing materials.

#### 3.4.2. Analysis of Effective Calcium Content

Considering the crystal structure parameters [22,23,24], specific surface areas and solubility in water of the three calcium-bearing materials, the effective calcium content in aqueous solution was calculated respectively. The calculation results are shown in Table 1. The calculation method is as follows: 

*m*_0_ is calculated according to the solubility of each substance.

(2) $$s_{1}=s_{0}\times m_{0}$$

(3) N=1s2

(4) $$n_{1}=n_{0}\times N$$

(5) $$n_{2}=n_{1}\times s_{1}$$

$n_{3}$ is calculated based on solubility of each substance.

(6) $$n_{4}=n_{2}+n_{3}$$

where m_0_ is the mass of solid phase in solution, g; s_0_ is the specific surface area of substance, m^2^/g; s_1_ is the surface area of solid substance in solution, m^2^; s_2_ is the cross-sectional area of a crystal cell, m^2^; N is the number of crystal cells per unit area; n_0_ and n_1_ are the calcium content of per crystal cell and per unit area of substance, respectively, mmol; n_2_ is the surface calcium content of solid phase in solution, mmol; n_3_ is the free calcium content in solution, mmol; n_4_ is the effective calcium content in solution, mmol. 

As can be seen from Table 1, the effective calcium content of CaO, CaF_2_ and CaCO_3_ in aqueous solution of 20 mL is in flowing descending order: 43.70 × 10^−2^, 9.81 × 10^−2^, 3.03 × 10^−2^ mmol. The As(V) adsorption quantity of CaO, CaF_2_ and CaCO_3_ are 2.00, 1.10 and 0.63 mg/g, corresponding to their effective calcium content in aqueous solution, indicating that the As(V) removal capacity of calcium-bearing materials is related to the effective calcium content in aqueous solution. However, the absolute value of the effective calcium content and adsorption quantity of CaO, CaF_2_ and CaCO_3_ in aqueous solution did not increase by a certain proportion, indicating that the types of anions in calcium materials would affect the chemical binding abilities of calcium ions and arsenate anions. The main As(V) adsorption mechanism of CaO, CaF_2_ and CaCO_3_ was chemical adsorption. On the one hand, arsenate anions bind to Ca^2+^ on the surface of the calcium-bearing materials; On the other hand, arsenate anions directly combine with free Ca^2+^ in solution to form arsenate precipitates. Thus, for calcium-bearing materials with simple components, choosing materials with high effective calcium content is more beneficial to the removal of As(V).

### 3.5. Chemical Stability of Three Calcium-Bearing Materials before and after Adsorption of As(V) 

**Figure 6:**
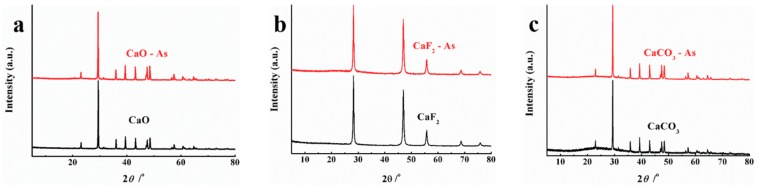
The XRD patterns of (**a**) CaO, (**b**) CaF_2_ and (**c**) CaCO_3_ before and after adsorption of As(V).

The chemical stability of adsorbents mainly depends on whether it’s crystal form changed or not. Therefore, crystal forms of CaO, CaF_2_ and CaCO_3_ were characterized by XRD before and after the adsorption of As(V), and the results are shown in Figure 6. From Figure 6 we can see that no excess emerged in the peaks, comparing pre- and post-adsorption of As(V), indicating all the three calcium-bearing materials exhibited good chemical stability under laboratory operating conditions. 

## 4. Conclusions

In conclusion, not only were the capabilities of removing As(V) by CaCO_3_, CaF_2_ and CaO compared according to removal rates and adsorption rates, but also As(V) removal mechanisms of the three absorbents were analyzed based on calculation of the materials’s crystal structures and specific surface areas. The results exhibit that (1) CaO has the best removal capacity on As(V), followed by CaF_2_ and CaCO_3_; (2) For As(V) aqueous solution with initial concentration of 0.2 mg/L, CaO and CaCO_3_ can reach to the adsorption equilibrium in 10 min, while CaF_2_ takes 60 min to reach the adsorption equilibrium. Especially, CaO can meet the requirement of arsenic limit (0.01 mg/L) in China’s drinking water sanitation standards, and it is expected to be an efficient adsorbent for the drinking water treatment with arsenic; (3) The ability of removing As(V) by CaO, CaF_2_, and CaCO_3_ is mainly related to the effective calcium content in aqueous solution; (4) CaO, CaF_2_, and CaCO_3_ exhibit great chemical stability after adsorption of As(V). The study will provide the basis for selecting high-effective calcium-bearing materials for the field of arsenic removal in aqueous solutions.

## Figures and Tables

**Table 1:** Effective calcium content of the three calcium-bearing materials.

Material Names	m_0_ (g)	n_1_ (10^−^^2^ mmol)	n_2_ (10^−^^2^ mmol)	n_3_ (10^−^^2^ mmol)	n_4_ (10^−^^2^ mmol)
CaO	0.17	2.89	1.69	42.00	43.70
CaF_2_	0.20	2.24	9.43	0.38	9.81
CaCO_3_	0.20	3.14	2.90	0.13	3.03

Note: The above calculation results are based on the aqueous solution volume of 20 mL.
